# IVIVC Assessment of Two Mouse Brain Endothelial Cell Models for Drug Screening

**DOI:** 10.3390/pharmaceutics11110587

**Published:** 2019-11-08

**Authors:** Ina Puscas, Florian Bernard-Patrzynski, Martin Jutras, Marc-André Lécuyer, Lyne Bourbonnière, Alexandre Prat, Grégoire Leclair, V. Gaëlle Roullin

**Affiliations:** 1 Faculty of Pharmacy, Université de Montréal, CP6128 Succursale Centre-ville, Montreal, QC H3C 3J7, Canada; ina.puscas@umontreal.ca (I.P.); florian.bernard@umontreal.ca (F.B.-P.); martin.jutras@umontreal.ca (M.J.); 2 Department of Neuroscience, Faculty of Medicine, Université de Montréal and Centre de Recherc and du CHUM (CRCHUM), Montréal, QC H2X 0A9, Canada; marc-andre.lecuyer@mail.mcgill.ca (M.-A.L.); lyne.bourbonniere.chum@ssss.gouv.qc.ca (L.B.); a.prat@umontreal.ca (A.P.); 3 Centre for Biostructural Imaging of Neurodegeneration, Institute for Multiple Sclerosis Research and Neuroimmunology, University Medical Center Göttingen, 37075 Göttingen, Germany

**Keywords:** mouse brain microvascular endothelial cells, primary cell culture, bEnd.3 cell line, IVIVC, blood-brain barrier

## Abstract

Since most preclinical drug permeability assays across the blood-brain barrier (BBB) are still evaluated in rodents, we compared an in vitro mouse primary endothelial cell model to the mouse b.End3 and the acellular parallel artificial membrane permeability assay (PAMPA) models for drug screening purposes. The mRNA expression of key feature membrane proteins of primary and bEnd.3 mouse brain endothelial cells were compared. Transwell^®^ monolayer models were further characterized in terms of tightness and integrity. The in vitro in vivo correlation (IVIVC) was obtained by the correlation of the in vitro permeability data with log BB values obtained in mice for seven drugs. The mouse primary model showed higher monolayer integrity and levels of mRNA expression of BBB tight junction (TJ) proteins and membrane transporters (MBRT), especially for the efflux transporter Pgp. The IVIVC and drug ranking underlined the superiority of the primary model (r^2^ = 0.765) when compared to the PAMPA-BBB (r^2^ = 0.391) and bEnd.3 cell line (r^2^ = 0.019) models. The primary monolayer mouse model came out as a simple and reliable candidate for the prediction of drug permeability across the BBB. This model encompasses a rapid set-up, a fair reproduction of BBB tissue characteristics, and an accurate drug screening.

## 1. Introduction

Research and drug discovery programs for central nervous system (CNS) therapeutics are among the most challenging and expensive in the pharmaceutical research field. This is mainly due to the high complexity of the brain structure, the side effects caused by CNS drugs, and their poor blood-brain barrier (BBB) permeation [1]. The BBB mostly consists of endothelial cells (ECs) tightly embedded in a continuous and uniform monolayer, forming a physical, transport, and metabolic barrier to all blood-borne molecules [2]. Thus, 100% of the passage of large drug molecules and more than 98% of smaller ones are blocked at the BBB [3]. Lipophilic molecules with a molecular weight lower than 400 Da are known to reach the brain parenchyma by passive diffusion, which postulates their passage through the cellular layer. The bypass between two adjacent cells, i.e., the paracellular route, is available to small hydrophilic molecules, such as sucrose or mannitol [4]. The active passage through the BBB uses a carrier transporter to move molecules from the blood to the brain, once the recognition of the substrate by its transporter occurs [5]. As such, in the early stages of drug discovery, in silico, in vitro, and in vivo tests are used to predict the BBB permeability and select the best drug candidates, with the aim to enhance their odds of success [6].

In silico models are computational mathematical simulations based on physicochemical properties such as molecular weight, lipophilicity, solubility, and number of hydrogen bonds [7]. These models constitute an excellent initial screening, enabling a rapid selection of drug candidates suitable for in vitro testing. The advent of machine learning and efforts to take into account efflux and uptake transports will definitely enhance the reliability of these models.

On the other hand, in vitro models offer a rapid, non-expensive high-throughput drug screening. These models are designed using primary, immortalized cell lines or even stem-derived cells in mono-, bi-, or tri-culture setups made from ECs along with astrocytes, pericytes, neurons, or microglia [8]. 3D microfluidic dynamic models, 3D collagen matrix models, 3D flow systems [9] or BBB spheroids [10] are designed to reproduce the BBB microenvironment and cell-cell communication. However, the complexity and difficult reproducibility of these model designs compromise their suitability as BBB drug screening platforms. On the contrary, static monolayer in vitro models represent a fair compromise between availability of cell material and expression of BBB membrane transporters (MBRT) and tight junction (TJ) proteins. Thus, adoption of in vitro models offering meaningful and relevant insights during early stages of pharmaceutical drug development could contribute to improve selection success rates, as well as reducing excessive animal testing during later stage of preclinical phase [11].

Permeability assays are important, not only for CNS leads, but for all newly synthetized chemical entities, as peripherally active compounds could affect the CNS and cause unwanted neurological side effects [12]. Consequently, a reliable in vitro model for the screening of large libraries of chemical compounds is needed. The biorelevance of an in vitro BBB model is frequently evaluated by measuring two parameters. First, the tightness of the model is assessed by the transendothelial electrical resistance (TEER). A low TEER value is linked to an increase in passive permeability [13]. The second parameter is the expression of the efflux transporter MDR1 (multidrug resistance 1 protein), also named the p-glycoprotein (Pgp). This protein is the principal reason of many drug development failures, as it is known to actively pump out small lipophilic drugs from the cytoplasm of endothelial cells to the blood circulation, thus preventing compounds to reach the brain parenchyma. Both parameters play key roles in the permeability of drug molecules across the BBB; however they are not the only ones [14]. In addition to TEER measurements, we chose to assess major BBB TJ proteins, namely claudin-5 (CL-5), zonula occludens 1 (ZO-1) and occludin (OCL). BBB is also known to express high levels of efflux transporters, such as Pgp and the breast cancer resistance protein (BCRP). Finally, solute-like carriers, a family of influx transporters including GLUT-1 and LAT-1, are also overexpressed in BBB endothelial cells. Currently, in order to screen BBB drug candidates, pharmaceutical industries mostly use in vitro models which were first described to test intestine permeability. They are mainly the totally acellular parallel artificial membrane permeability assay (PAMPA) or non-BBB cellular models, such as the Caco-2 cells, issued from an heterogeneous human epithelial colorectal adenocarcinoma, or the MDCK-MDR1 cell line, a canine renal epithelial model. [15]. PAMPA, a non-cellular high-throughput, low-cost technique, only predicts drug permeation mediated by passive diffusion. Caco-2 and MDCK-MDR1 cell models, known for their high TEER and expression of Pgp protein, are, however, not brain-derived, thus morphologically different from cerebral endothelial cells. Indeed, drug transport across the BBB is inversely correlated to the enhanced presence of specific TJ and specialized MBRT proteins; however those proteins are less expressed in the previously mentioned cell models than in BBB endothelial cells [16]. Those mentioned characteristics should be present in in vitro models meant for BBB drug screening.

As far as brain cells are concerned, the most well-established cell lines for designing in vitro BBB models are the human microvascular endothelial cell line hCMEC/D3, the rat endothelial cell line RBE4, and the mouse brain microvascular endothelial cell line bEnd.3 [17]. These immortalized cell lines are commercially available tools, they proliferate well, and are easy to handle. On the other hand, primary BBB ECs, originating from brain microvessels, are thought to preserve most of in vivo characteristics prone to be lost during the immortalization process. For instance, the hCMEC/D3 cell line is known to present lower levels of claudin-5 expression as well as other tight junctions, overall leading to leaky models [13]. Primary cells are often isolated from bovine, porcine, rat, mouse, or human brains [13]. Human primary cells are not readily available, especially from healthy brain tissues. Induced pluripotent stem cells (iPSC) are proposed as a theoretically unlimited renewable source of human brain cells. However, the generation of purified brain-derived ECs from iPSC is a few weeks long process using complex, expensive differentiation medium. Overcoming these issues will guarantee the widespread use of iPCS in basic research [18]. Bovine and porcine EC isolations yield a large amount of cell material. Yet, the housing and maintenance of those animals in lab research are complicated and so, the brains are usually acquired from slaughterhouses [19]. However, in order to be used in research work, animals should entirely comply with disease and safety controls. On the contrary, rats and mice are ubiquitous, well-established small laboratory animals, readily available to most labs worldwide. Mice also display another advantage over human models as several genetically-modified mice have already been produced, including humanized mice expressing human transporters [20]. Due to their rapid reproduction and maturation to adulthood, they are both good choice models to retrieve primary cells.

In this study, we hypothesized that using primary mouse cells may offer a closer representation to the major BBB characteristics, due to better preserved expression of transporters and TJ proteins implicated in drug trafficking across the BBB. We supposed that this primary model could still allow for a rapid screening of drug candidates. without increasing the overall complexity of evaluating drug permeability. To this end, two mouse brain ECs in vitro models were compared and evaluated for their relevance as BBB drug screening models. One of them used freshly-isolated primary cells and the second one used the immortalized bEnd.3 cells. Ultimately, the cellular models were measured up with the early-stage pharmaceutical drug screening tool PAMPA-BBB, a non-cellular model consisting of a polymeric membrane soaked with porcine brain phospholipids (PBL). Cellular models were designed in a static, two-compartment monolayer model. MBRT and TJ protein expressions were assessed and compared between the cellular models. Ultimately, the permeability data of seven drugs obtained in the three models were correlated with the in vivo permeation coefficient obtained in mice in order to determine which model best fitted the in vitro/in vivo correlation (IVIVC).

## 2. Materials and Methods

### 2.1. Chemical and Supplies

Dulbecco’s Modified Eagle Medium high glucose (DMEM), bovine serum albumin fraction V (BSA), Dulbecco’s Phosphate Buffered Solution (DPBS), Fetal Bovine Serum (FBS), Penicillin Streptomycin 100× (Pen/Strep) and Extreme-DMEM (X-DMEM) were purchased from Wisent (Saint-Jean-Baptiste, QC, Canada). Type II collagenase and type I deoxyribonuclease (DNase) were purchased from Worthington Biochemical Corp. (Lakewood, NJ, USA). Collagenase/dispase, type IV collagen from human placenta (type IV collagen), hydrocortisone, Insulin/Transferrin/Sodium Selenite supplement 100× (ITS), puromycin dihydrochloride from *Streptomyces alboniger*, sodium fluorescein (NaFl), fluorescein isothiocyanate-dextran (FITC-Dextran) and metoclopramide hydrochloride were purchased from Sigma-Aldrich (Oakville, ON, Canada). Basic Fibroblast Growth factor (bFGF), trypsin-EDTA 0.25% and 0.5% were purchased from Thermo Fisher Scientific (Burlington, ON, Canada). Zonula occludens-1(ZO-1) and Claudin-5 (CL-5) polyclonal antibodies, goat anti-rabbit immunoglobulins conjugated with Alexa Fluor^®^ 488 and Slow Fade Diamond Antifade Mountant with DAPI were purchased from Invitrogen™ (Burlington, ON, Canada). Sodium heparin were purchased from Fisher Scientific (Ottawa, ON, Canada). Percoll was purchased from GE HealthCare Bio-Sciences (Baie-d’Urfé, QC, Canada) and blank mouse (cd-1) plasma EDTA K2 were purchased from BioIVT (New York, NY, USA). Transwell^®^ Costar 24-well plates (Costar^®^: 3470; 6.5 mm inserts; polyester membrane; pore size: 0.4 µm; pore density, 4 × 106 pores/cm^2^) were purchased from Corning Inc. (Kennebunk, ME, USA). (3-(4,5-dimethylthiazol-2-yl)-5-(3-carboxymethoxyphenyl)-2-(4-sulfophenyl)-2*H*-tetrazolium) (MTS) cells proliferation kit were purchased from Abcam^®^ (Toronto, ON, Canada). Brain mouse endothelial cell line bEnd.3 (ATCC^®^ CRL-2299) was purchased from Cedarlane (Burlington, ON, Canada) and was used from passages 8 to 20.

### 2.2. Animals and Cell Culture

All animals (C57BL/6 mice) were obtained from Charles River (Senneville, QC, Canada) and thereafter bred at the Marcelle-Coutu animal facility, Université de Montréal; they were treated in compliance with the institutional requirements set out by the Comité de Déontologie de l’Expérimentation sur les Animaux (CDEA) of Université de Montréal, Montréal, Québec, Canada (approved protocols #17-078 and -079, granted on 22 September 2017, renewed on 28 September 2018). The animal protocols further complied with the health guide for the care and use of Laboratory animals (NIH Publications No. 8023, revised 1978).

#### 2.2.1. Primary Cell Isolation and Culture

Primary brain mouse endothelial cells (BMEC) were isolated from 5 to 8-week-old C57BL/6 mice, according to the protocol reported by Lécuyer et al. [21]. Typically, 20 female and male mice were euthanized under CO_2_. Brains were surgically removed and then meninges were rolled out prior to mechanical digestion. Mechanical digestion was performed by finely slicing brain tissues, followed by forcing the sliced tissues five times first through a 18-G needle then a 20-G one. The obtained homogenates were digested in DMEM containing 1.05 mg/mL type II collagenase and 58.5 U/mL type I DNase for 1 h 15 min at 37 °C on a benchtop shaker incubator (200 rpm). Collected pellets were then incubated with 20% (*w/v*) BSA diluted in DMEM and then centrifuged twice (1000× g, 20 min) in order to remove the neuronal myelin sheath. A second pellet digestion was performed in DMEM containing 1 mg/mL collagenase/dispase and 39 U/mL type I DNase for 1 h 15 min at 37 °C on a benchtop shaker incubator (200 rpm). The cluster separation and cellular contaminant removal were performed by the centrifugation of the pellet on 33% continuous Percoll gradient at 1000× g for 10 min, 4 °C. Percoll residues were removed by centrifuging the capillaries in DMEM at 800× g for 8 min. Afterwards, recovered capillaries were seeded on 6-well plates, previously coated for 4 h with type IV collagen 5 µg/cm^2^ reconstituted in DPBS. On the day one of the isolation, the capillaries were cultured in BMEC specific medium consisting in DMEM medium supplemented with 20% FBS, 1 × Pen/Strep, 1 ng/mL bFGF, 100 µg/mL sodium heparin, 1.4 µM hydrocortisone, 0.2% ITS and 10 µg/mL puromycin. Afterwards, cells were cultured in BMEC medium containing 4 µg/mL puromycin and the medium was changed every two days. Once cells reached 90–95% confluency, ECs were harvested by using trypsin-EDTA 0.5%. All experiments were performed with cells at passage 1.

#### 2.2.2. Cell Line Culture

bEnd.3 mouse endothelioma cell line was cultured in DMEM/10% FBS/1 × Pen/Strep at 37 °C in a humidified 5% CO_2_ incubator (Forma™ Steri-Cycle™ CO_2_, Thermo Fisher Scientific, Burlington, ON, Canada). The culture medium was changed every two days. When confluent, cells were harvested using trypsin-EDTA 0.25%. All experiments were performed on cells at passages 8 to 20.

#### 2.2.3. Morphological Cell Characterization

Cell status, rate growth and purity were routinely observed under an optical microscope, with 10× and 20× magnifications. Images were recorded using a ZEISS AXIOVERT S100 microscope (Carl Zeiss Microscopy LLC, Peabody, MA, USA) equipped with a Moticam 3+ camera (Moticam, Kowloon, Hong Kong) and processed with ImageJ software (version 1.51, National Institutes of Health, Bethesda, MD, USA).

#### 2.2.4. Puromycin Cell Viability Assay

Cell proliferation ability in presence of increasing puromycin concentrations was investigated on BMEC and bEnd.3 cells by a MTS assay. Briefly, 5 × 10^3^ cells were seeded in each well of a 96-well plate, in a final volume of 200 µL/well. Cells were allowed to adhere 24 h prior to replacing all culture media with 100 µL of media containing puromycin at concentrations ranging from 0.01 to 100 µg/mL. Cells were then incubated at 37 °C in a humidified 5% CO_2_ incubator for 24 h. Thereafter, 11 µL of the MTS reagent were added in each well and left to incubate for 3 h at 37 °C in standard culture conditions. The reduction of the MTS tetrazolium compound by viable cells generated a colored formazan product, soluble in cell culture medium, which was quantified by measuring the absorbance at 490 nm (Safire spectrophotometer, Tecan, Männedorf, Switzerland). Negative controls were untreated cells, whereas positive controls were cells treated with 0.1% of triton 100 × 1 h prior adding MTS. Cell viability was calculated as follows (Equation (1)):

% viable cells = (OD_assay_ − OD_medium_)/(OD_untreated cells_ − OD_medium_) × 100 (1)

where OD_assay_: absorbance in sample well; OD_medium_: absorbance of medium alone; OD_untreated cells_: absorbance of untreated cells.

### 2.3. Model Assembly

To build ECs models, cells were seeded inside the upper inserts of Transwell^®^ 24-well plates. Prior to seeding, the 6.5 mm polyester membranes [22] were coated with a type IV collagen solution (5 µg/cm^2^). After 4 h, excess collagen was removed. Cells were then seeded at 2 × 10^5^ cells/cm^2^ [23]. Primary ECs models were maintained in their specific culture medium and bEnd.3 models were maintained in DMEM/10% FBS/1 × Pen/Strep at 37 °C/5%CO_2_. Media were changed every two days. Under these conditions, the in vitro models were ready within 7 days.

#### 2.3.1. Evaluation of the Model Integrity

• Transendothelial electrical resistance (TEER) measurement

TEER measurements were used as a non-invasive and quantitative technique to evaluate the TJ dynamics, and thus the barrier formation. Prior to measurements, Transwell^®^ plates were equilibrated for 20 min at room temperature (RT). Then inserts were transferred in the Endohm-6 cup (World Precision Instruments, Sarasota, FL, USA) and the ohmic resistance was measured using the Millicell^®^ ERS voltmeter (Millipore, MA, USA). Model resistances were measured in their respective growing medium. TEER_total_ values were calculated by multiplying the obtained resistance (ohm) by the insert area (S, cm^2^), as in Equation (2). To calculate the TEER of the monolayer, the mean TEER value of cell-free inserts was subtracted to the TEER_total_, as in Equation (3):

TEER_total_ = Resistance (Ω) × S_insert_ (cm^2^) (2)

TEER_monolayer_ (Ω × cm^2^) = TEERt_otal_ − TEER_cell-free insert_ (3)

• Fluorescent marker permeability assay

Two fluorescent markers were used to evaluate model permeabilities: sodium fluorescein (NaFl, MW = 376 g/mol) and fluorescein isothiocyanate-dextran (FITC-Dextran, MW = 150 kDa). Monolayer inserts were transferred to a 24-well plate containing 600 µL of X-DMEM in the lower compartments. Media from the upper compartments were replaced by either 100 µL of 10 µg/mL NaFl or 1 mg/mL of FITC-Dextran in X-DMEM. Five time points were realized at 15, 30, 45, 60 and 75 min. Following each time sampling, inserts were transferred to a new 24-well plate containing fresh X-DMEM (600 µL/well). Samples concentrations were determined by fluorescence (Saffire, Tecan, Männedorf, Switzerland). Excitation/emission wavelengths were 485/520 nm and 492/518 nm for NaFl and FITC-Dextran, respectively. The fluorescent marker endothelial permeability coefficient (Pe) was calculated as fully detailed in Section 2.3.3.

#### 2.3.2. In vitro Drug Permeability Assay

In order to validate the primary cell and the bEnd.3 models, seven drug molecules with various physicochemical properties were chosen (Table 1). Chlorpromazine (D1, an antipsychotic agent) and midazolam (D2, a sedative drug), readily permeate through the BBB. Caffeine (D3) and theophylline (D4) are mild brain psychostimulants and so reach the brain tissue at a lesser extent. Verapamil (D5, a calcium-channel blocker), atenolol (D6, a cardioselective β-blocker) and tenoxicam (D7, an anti-inflammatory agent) are not normally distributed to the brain. Thus, this panel of drugs covers high, moderate and low BBB passage, as well as a Pgp substrate (verapamil, D5).

Inserts containing monolayer models were transferred to a 24-well plate containing 600 µL of X-DMEM in the lower compartments. Media from the upper compartments were replaced by 100 µL of the tested drug solubilized in X-DMEM (10 µM, *n* = 4/drug). Five time points were sampled at 15, 30, 45, 60 and 75 min. Collected samples were analyzed by LC-MS/MS, with metoclopramide hydrochloride as the internal standard. Details of the LC-MS/MS analysis are summarized in Table 2 and Section 2.6. *p* values were calculated as indicated in Section 2.3.3.

#### 2.3.3. Permeability Coefficient (Pe) Calculation

The Pe was calculated as previously stated in the work of Deli et al. (2005) [24] and Nakagawa et al. (2009) [23]. First the cleared volume (µL), corresponding to the tested molecule transport from the upper compartment to the lower compartment, was calculated from Equation (4):

Cleared volume (µL) = (C_lower compart._ × V_lower compart._)/C_upper compart_ (4)

with C_lower compart._ being the concentration of tested molecule in the lower compartment, V_lower compart._ the volume of the lower compartment (i.e., 600 µL), C_upper compart._ the concentration of the tested molecule in the upper compartment.

Then, the cumulative cleared volume at each time point (15, 30, 45, 60 and 75 min) was calculated. The product (PS) of the drug permeability by the insert area (0.33 cm^2^) was calculated as the slope of the plotting of cumulative volumes against time. The PS of the ECs monolayer were calculated using Equation (5).

1/PS_endo_ = 1/PS_total_ − 1/PS_insert_ (5)

where PS_endo_ is the product between the Pe of the ECs monolayer and the insert area (cm^3^/s); PS_total_ is the product between the Pe of the tested model and the insert area (cm^3^/s); PS_insert_ is the product between the Pe of the cell-free insert and the insert area (cm^3^/s).

Finally, the Pe of the ECs monolayer was calculated as shown in Equation (6):

Pe (cm^2^/s) = PS_endo_/S_insert_ (6)

#### 2.3.4. Model Characterization

• Immunostaining

To characterize the monolayer model integrity, 7-day old ECs monolayers were stained for junctional proteins with ZO-1 and CL-5 polyclonal antibodies. All antibody dilutions were performed in X-DMEM (primary antibodies 1:100 dilution; secondary antibody: 1:200 dilution). First, inserts were washed in DPBS and cell monolayers were fixed and permeabilized for 15 min at room temperature (RT, 21 ± 1 °C) with cold methanol (−20 °C). To reduce background interference, the excess protein-binding sites in cells were blocked with 3% BSA for 1 h at RT or overnight at 4 °C. Incubations with the anti-ZO-1 and anti-CL-5 primary antibodies were performed in the same conditions as the BSA blocking step. Finally, cells were incubated with the secondary antibody Alexa Fluor 488-conjugated goat anti-rabbit for 1 h at RT. Between incubations, inserts were washed thrice, 5 min each, with PBS on a benchtop shaker incubator (100 rpm). Next, membranes with the monolayers were cut off from the inserts and placed on lamellae for microscopic examination, with the cell monolayer facing up. Nuclei were stained with Slow Fade Diamond Antifade Mountant with DAPI and samples were examined using a fluorescence microscope Olympus IX81 (Olympus, Waltham, MA, USA), equipped with a Retiga 2000R CCD camera (QImaging, Surrey, BC, Canada). Images were acquired with a MetaMorph Advanced software (v. 7.8.9.0, Molecular Devices, LLC, San José, CA, USA) and processed with ImageJ software (v1.51, National Institutes of Health, Bethesda, MD, USA).

• Quantitative polymerase chain reaction (qPCR)

Total RNA was extracted from lyzed BMEC and bEnd.3 cells. Cells were either obtained from culture dishes (cell characterization) or inserts (monolayer characterization). First, samples were purified from genomic DNA on cDNA eliminator mini spin columns (Qiagen, Hilden, Germany). Then mRNA was extracted on RNase mini spin columns (Qiagen, Hilden, Germany) following the manufacturer guidelines. The obtained RNA was reverse-transcribed into cDNA using a high-capacity cDNA Reverse transcription kit (Thermo Fisher Scientific, Burlington, ON, Canada) and amplified in a thermocycler (Analytic Jena, Jena, Germany). All primers used in this study are listed in Table 3. The mouse hypoxanthine-guanine phosphoribosyltransferase (HPRT) enzyme gene was used as the endogenous control. Each sample was prepared in triplicate, while non-reversed RNA and no DNA template served as negative controls. The qPCR reactions were performed by 40 cycles of 95 °C for 5 s and 60 °C for 30 sec, using the ViiA7 or QuantStudio 7 Flex QPCR system (Thermo Fisher Scientific, Burlington, ON, Canada). The comparative CT method (2^–ΔΔCT^ method) was used to calculate the relative expression level of each target gene. Data were analyzed as the fold change in gene expression using the GraphPad Prism^®^ software (v. 7.01, GraphPad Software, San Diego, CA, USA).

• Droplet digital polymerase chain reaction (ddPCR)

ddPCR was performed on cDNA. The ddPCR system QX200 Droplet Digital PCR (Bio-Rad, Hercules, CA, USA) was used to fractionate samples into ~20,000 droplets. Amplification was performed in a 20-μL multiplex reaction containing 1 ng of purified cDNA, 800 nM of primers and 250 nM of probes, 2× ddPCR Supermix for probes (no UTP). Samples were subjected to droplet generation by an automated droplet generator and later end-point PCR was performed. Cycling steps for the ddPCR were as follows: initially an enzyme activation at 95 °C for 10 min followed by 50 cycles of denaturation and annealing (each cycle at 95 °C for 30 s; 58 °C for 1 min; 72 °C for 30 s) and finally enzyme deactivation at 98 °C for 10 min. Finally, droplets were read on a droplet reader and data were analyzed using the QuantaSoft™ Software which determines the numbers of droplets being positive and negative for each fluorophore in each sample. The fraction of positive droplets was then fitted to a Poisson distribution in QuantaSoft™ Software to determine the absolute number of copies in units of copies per μL.

### 2.4. PAMPA Model

Twenty mg of porcine brain lipids (PBL) were added in a round-bottom flask and allowed to evaporate for 10 min on a rotary evaporator. The resulting lipid film was then solubilized with 1 mL of dodecane, to reach a PBL concentration of 20 mg × mL^−1^. The PAMPA setting consisted of two parts, the acceptor plate equipped with 0.45 µm PVDF filters and the donor bottom 96-well plate. Prior to starting the assay, the PVDF filters were coated with 10 µL of the 20 mg × mL^−1^ PBL solution and then dried for 10 min at RT. As the PAMPA model used in this work was designed using PBL, it will be referred as PAMPA-BBB throughout this research article.

Standard stock solutions of verapamil (15 mM), midazolam (53 mM) and tenoxicam (50 mM) were prepared in DMSO, whereas those of chlorpromazine (28 mM), caffeine (26 mM), atenolol (3.7 mM) and theophylline (46 mM) were prepared in PBS at pH = 7. Thereafter, the seven drug solutions were combined and further diluted with PBS in order to obtain a working solution of 50 µM (0.23% DMSO), named the cassette. Its stability was established at 5 ± 2 °C and −80 ± 2 °C for 16 and 31 days, respectively.

The acceptor plate containing 300 µL/well of PBS was placed on top of the donor plate containing 300 µL/well of the cassette, thus creating a ‘sandwich’ layout with PBL-coated filters in between. The setting was incubated at 37 °C for 18 h under shaking at 50 rpm (MaxQ 4000, Barnstead/Lab-line^®^ Melrose Park, IL, USA). Donor and acceptor samples were analyzed by LC-MS/MS (Section 2.6), using metoclopramide (250 mM) as the internal standard (ISTD).

Drug permeability, namely Papp, was calculated with Equation (7), according to Balimane et al. [25]:

Papp = V × dC/A × C0 × dt = Q/t × A × C_0_ = rC_r_ × V_r_/t × A × rC_0_ (7)

where Papp: apparent permeability (cm·s^−1^); Q: quantity of diffused drug at the end of the assay (mol); t: duration of the assay (s); A: membrane surface (cm^2^); rC_0_: relative concentration (signal ratio of the AUC_drug_ on the AUC_ISTD_) in the donor compartment; rC_r_: relative concentration (signal ratio of the AUC_drug_ on the AUC_ISTD_) in the receiver compartment; V_r_: volume of the receiver compartment (cm^3^).

### 2.5. Animal Studies

In vivo studies of drug brain permeability were performed in 5 to 8-week-old C57BL/6 mice, weighting between 17 and 33 g. Each drug was formulated in an adequate vehicle in order to ensure total dissolution and a safe, non-painful injection (Table 4). Drug solutions were passed through a sterile 0.22-µm filter to warranty sterility, prior to being administered intravenously as a bolus in the tail vein at a dosage of 5 mL/kg. Animals (*n* = 4 mice/drug) were injected and sacrificed 2 h later. To that purpose, mice were first anesthetized under isoflurane. Then total blood was withdrawn intracardiacally and brains were removed after decapitation. The plasma separation was performed by centrifugation at 3000 rpm/4 °C/20 min. Plasma samples were either immediately analyzed or stored at −80 °C until further analysis. Prior to the LC-MS/MS analysis, fresh brains were crushed using a Polytron homogenizer (Kinematica GMBH, Luzern, Switzerland) and underwent protein precipitation by adding a mixture of acetonitrile/water (50:50). Finally, brains were diluted with a blank brain suspension and plasma samples were diluted with blank mouse (cd-1) plasma EDTA K2 (BioIVT, Westbury, NY, USA). Collected samples were analyzed by LC-MS/MS, with metoclopramide as ITSD (Table 2 and Section 2.6). The log of the brain-to-blood ratio was calculated according to Bickel’s report [26], as in Equation (8):

log BB = log (C_brain_/C_blood_) (8)

where C_brain_ is the concentration quantified in the brain and C_blood_ the concentration quantified in the plasma.

#### In Vitro/In Vivo Correlation (IVIVC)

The correlation between log BB and log Papp or log Pe was established using the GraphPad Prism^®^ software (v. 7.01) by plotting the average log Papp or log Pe value of each drug against its mean in vivo counterpart (log BB). Coefficient of determination (r^2^) was then calculated using linear regression. In order to establish the drug ranking for a given model, each drug log value (log value_drug_) was subtracted by the lowest drug log value (log value_min_) and then divided by the difference between the highest (log value_max_) and lowest (log value_min_) values in the distribution, as in Equation (9):

normalized log value_drug_ = (log value_drug_ − log value_min_)/(log value_max_ − log _valuemin_) (9)

This normalization of log values was used to preserve distribution and order intact for each model.

### 2.6. LC-MS/MS Drug Analysis

The gradient LC-MS/MS system consisted of an Agilent Technologies HPLC 1100 Series (Mississauga, ON, Canada) equipped with a degassing system (G1379A), a binary pump (G1312A), a refrigerated autosampler (G1367A) and a column oven (G1316A). The detector was an API4000 Q TRAP™ hybrid triple quadrupole/linear ion trap mass spectrometer (AB Sciex, Framingham, MA, USA) with a turbo electrospray ionisation source (ESI). Chromatographic and detection conditions are detailed in Table 2. Mass spectrometry (MS) conditions for all drugs were set at infusing 1 µM of the pooled test solutions at 10 µL/min while the dwell times were set at 70 ms. Chromatography was performed using a YMC-Pack ODS-AQ column (5 µm, 3.0 × 50 mm; YMC America Inc., Allentown, PA, USA). Sample injection volume was 4 µL and the LC flow rate was 0.75 mL·min^−1^. Mobile phase (A) was milliQ water with 0.2% formic acid (FA) and mobile phase (B) was acetonitrile with 0.1% FA. The gradient elution started with a 20-s hold at 2% mobile phase (B), followed by a 3.5-min ramp to 100% mobile phase (B). Next, there was a 1.1-min stabilization at 100% (B) and a fall in 10 s at 2% (B) followed by a re-equilibration at 2% (B) for 2 min. The mass spectrometer and peripherals (pumps and autosampler) were all controlled by Analyst^®^ software (v1.6.2; AB Sciex, Concord, ON, Canada).

### 2.7. Statistical Analysis

All data are presented as means ± standard deviations and differences were considered statistically significant at *p* < 0.0332 using the unpaired t-test with Welch’s correction (*p* ≥ 0.0332: non-statistically significant (ns), * *p* < 0.0332, ** *p* < 0.0021, *** *p* < 0.0002, ND: not detected). The puromycin cell viability assay data were analyzed using a 2-way ANOVA test and differences were considered statistically significant at *p* < 0.0332.

## 3. Results

### 3.1. Endothelial Cells—Basal Characterization

#### 3.1.1. Morphology of Mouse Brain Endothelial Cells

Primary brain endothelial cell isolation from mice provides cellular confluent layers in 5 to 8 days. ECs were identifiable by their spindle shape and by their specific proliferating pattern in a dense homogeneous monolayer. Their nuclei were well-defined, in contrast with their membranes which were more difficult to observe. When proliferating, they multiplied while remaining in contact with other ECs but no overlapping phenomenon was observed. A concentric pattern was observed around the brain capillaries from which duplicated cells emerged.

Culture of the bEnd.3 cell line (passages 8 to 20) allowed obtaining confluent monolayers in 2 to 4 days. As for primary cells, the monolayers were composed of spindle-shaped cells, with visible dense nuclei and cell membranes difficult to detect. These cells also grew in a dense, non-overlapping monolayer. It was impossible to differentiate cells according to their origin by a simple microscopic observation of their morphology (Figure S1).

#### 3.1.2. Specific mRNA Gene Expression in Brain Mouse Endothelial Cells

Gene expression of main MBRT and TJ proteins was quantified using the qPCR technique (Table 3). Thus, the mRNA expression of two efflux transporters (Pgp and BCRP), two nutrient transporters (GLUT-1 and LAT-1) and three TJ proteins (ZO-1, OCL, CL-5) was analyzed. Prior to qPCR, the mRNA quality was assessed using the Agilent 2100 bioanalyzer system and RIN for all samples were within 8.9–9.9.

**Figure 1:**
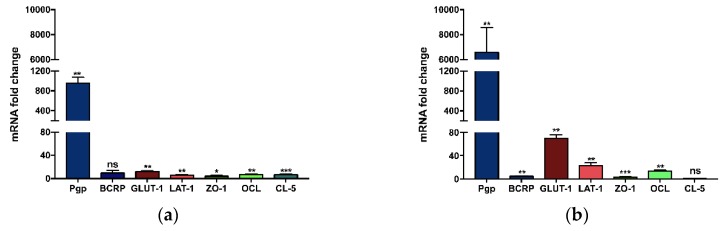
mRNA expression fold-change between BMEC and bEnd.3 cells, the latter used as the calibrator. Both cell monolayers were either grown in simple DMEM medium (**a**) or in specialized BMEC medium (**b**). Relative gene expression was calculated using the 2^−ΔΔCt^ method, with HPRT as the housekeeping gene and the bEnd.3 cell line as the calibrator. Bars indicate the average mRNA fold change ± SD (*n* = 3). Data were analyzed using an unpaired *t* test with Welch’s correction (non-statistically significant (ns): *p* ≥ 0.0332, * *p* < 0.0332, ** *p* < 0.0021, *** *p* < 0.0002).

Since cells were grown in specific media according to their origin, the first step consisted of comparing gene expressions of primary BMECs grown in their own specialized culture medium to that of bEnd.3 cells grown in DMEM/10% FBS/1×Pen/Strep medium (Figure 1a). In these conditions, and after normalizing results, BMEC were found to express significantly higher gene expression levels for most of the tested proteins compared to bEnd.3 cells (955-fold increase for Pgp, 12-fold for GLUT-1, 6-fold for LAT-1, 4-fold for ZO-1, 7-fold for OCL, and 6-fold for CL-5, respectively). No significant difference was noticed for BCRP mRNA coding gene (Figure 1a).

Next, to evaluate the influence of the growth medium on the gene expression, the relative mRNA gene expression of BMEC was compared to that of bEnd.3 cells, both grown in the specialized BMEC culture medium. BMEC medium, in addition to basic elements, is composed of bFGF, heparin, hydrocortisone and ITS, all absent of the bEnd.3 medium, and all favoring endothelial cell growth and monolayer tightness. Normalized mRNA gene expression levels were statistically greater in BMECs than in bEnd.3 (6576-fold increase for Pgp, 4-fold for BCRP, 69-fold for GLUT-1, 22-fold for LAT-1, 3-fold for ZO-1, and 13-fold increase for OCL respectively). No significant difference was noticed for CL-5 (Figure 1b)

The same tendency, i.e. BMEC expressing far more mRNA than b.End3 cells, was observed when the samples were analyzed by ddPCR: (2319-fold for Pgp, 3-fold for BCRP, 11-fold for GLUT-1, 12-fold for LAT-1, 2-fold for ZO-1, and 9-fold for OCL and 0.3-fold increase for CL-5), as shown in Figure S2.

#### 3.1.3. Puromycin Viability Assay

**Figure 2:**
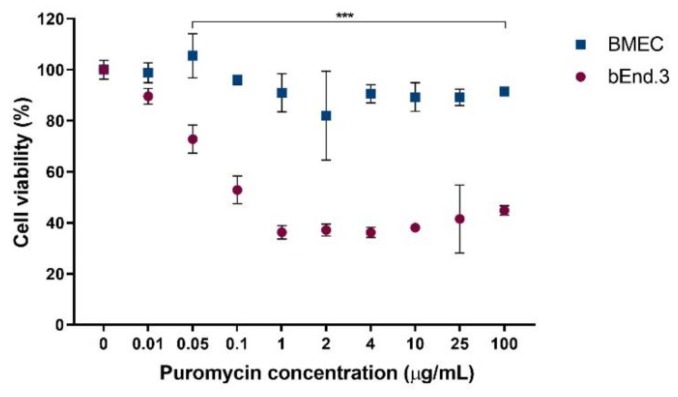
Effect of puromycin on BMEC (blue) and bEnd.3 cell (red) viability. Data were analyzed using a 2-way ANOVA test (*** *p* < 0.0001). Points represent the average cell viability ± SD (*n* = 6).

Puromycin is a Pgp substrate used for ECs selection from freshly-isolated brain capillaries [27]. Used at 4–10 µg/mL, cell cultures are progressively enriched in ECs, as only these cells display enough Pgp to protect themselves from puromycin toxicity. In order to assess if the difference in Pgp mRNA expression between the tested cells could be directly interpreted as a lower amount of protein present in the bEnd.3 cell line compared to BMECs, a cell viability assay was performed in the presence of increasing puromycin concentrations (0–100 µg/mL). From puromycin concentrations as low as 0.05 µg/mL, the bEnd.3 cell viability was significantly reduced (by ~40–50%) compared to untreated cells, whereas BMEC viability remained between 80–110%, whatever the tested concentration (Figure 2).

### 3.2. Monolayer Models and Subsequent Characterization

#### 3.2.1. TEER and Pe of Fluorescent Markers

Primary BMEC and bEnd.3 cells were turned into in vitro models when cultured on porous polymeric membranes separating two compartments (Transwell^®^ plates). Cells confluence was observed on day 7. Two techniques were used to compare the effectiveness of the BMEC and the bEnd.3 models to form a tight monolayer. First, TEER calculations were performed on days 2, 3, 4. On day 7, monolayer tightness was evaluated using TEER calculation and, additionally, by measuring the permeability of two fluorescent markers. Both these markers are hydrophilic molecules with different sizes (NaFl: 360 Da, FITC-D: 150 kDa) used to evaluate the paracellular permeability.

**Figure 3:**
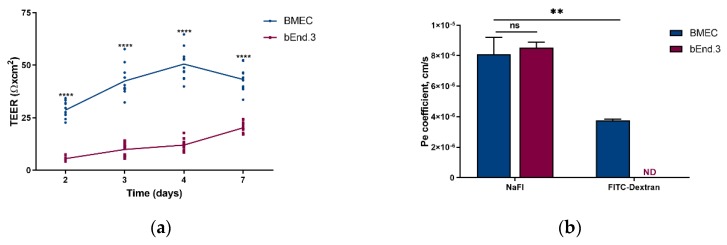
(**a**) Transendothelial electrical resistance (TEER, expressed as Ω × cm^2^) and (**b**) endothelial Pe for sodium fluorescein (NaFl) and FITC-dextran (Pe, expressed in cm/s) of the blood–brain barrier models built from mouse primary brain endothelial cells (BMEC, blue) and from mouse brain endothelial cell line (bEnd.3, red) at day 7. All data are presented as means ± SD (*n* = 12 for TEER, *n* = 4 for Pe). Statistical analysis: unpaired t test with Welch’s correction (ns: *p* ≥ 0.0332, **** *p* < 0.0001, ND-not detected).

TEER for the BMEC model showed 2-fold higher values compared to those calculated for the bEnd.3 model. This significant difference was observed from day 2 to 7 (Figure 3a). Thus, on day 7, the primary model displayed a TEER value of 80 ± 10 Ω × cm^2^ while the bEnd.3 model showed a TEER of 40 ± 4.5 Ω × cm^2^. Moreover, a significant increase was observed for the BMEC model when comparing the TEER values at days 3, 4 and 7 with the TEER value at day 2.

NaFl permeability assay resulted in a similar Pe for the BMEC model (8.1 ± 1.1 × 10^−6^ cm/s) and the bEnd.3 one (8.5 ± 0.4 × 10^−6^ cm/s) (Figure 3b). FITC-dextran was not detected in the lower compartment of the bEnd.3 monolayer after 75 min, while the BMEC model yielded a Pe of 3.8 ± 0.1 × 10^−6^ cm/s.

#### 3.2.2. Influence of the Support Material on the Gene Expression of Brain Endothelial Monolayers

In order to ensure that the differences observed between both types of ECs were not related to any specific affinity for their culture support, the qPCR technique was used to compare the relative mRNA gene expression levels of specific proteins.

First, brain ECs grown in a culture flask were compared to those grown on the permeable polyester membrane of the Transwell^®^ inserts (as used in the BBB model setup).

**Figure 4:**
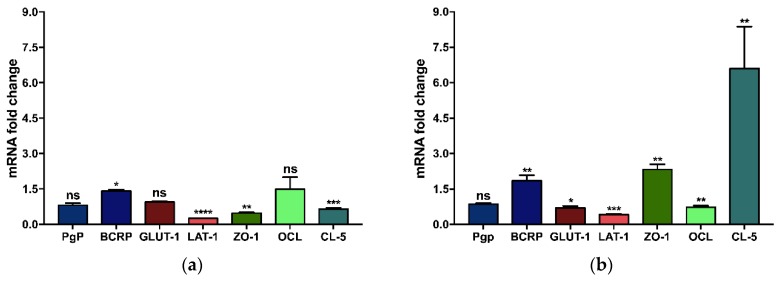
Differences in the fold-change of gene expression between (**a**) BMEC and (**b**) bEnd.3 cells grown on a polyester membrane insert filter compared to cells grown on plastic cell culture flask. Relative gene expression was calculated using the 2^−ΔΔCt^ method, with HPRT as the housekeeping gene and cells grown on the plastic cell culture flask as the calibrator. Data were analyzed using an unpaired *t* test with Welch’s correction (ns: *p* ≥ 0.0332, * *p* < 0.0332, ** *p* < 0.0021, *** *p* < 0.0002, **** *p* < 0.0001). The bars indicate the average mRNA fold change ± SD (*n* = 3).

Primary BMECs grown on the polyester membrane insert filter displayed statistically significant mRNA fold change expression for BCRP (1.4-fold increase) and LAT-1, ZO-1, and CL-5 (4-, 2- and 1.5-fold decreases, respectively) compared to BMEC grown in a culture flask. There were no significant differences for Pgp, GLUT-1 and OCL mRNA expression (Figure 4a). The complementary ddPCR analysis highlighted that mRNA expression for ZO-1 and CL-5 was decreased by 1.5- and 1.2-fold, respectively, when cells were cultured on the polyester membrane, while no difference was noticed for Pgp, GLUT-1 and OCL mRNA expression (Figure S3a).

For bEnd.3 cells grown on the polyester membrane insert filter, the mRNA fold change expression for BCRP, ZO-1, and CL-5 showed a 1.8-, 2.3- and 6.6-fold increase, respectively. For GLUT-1, OCL, and LAT-1, the gene expression presented a 1.4-, 1.4-, and 2.5-fold decrease, respectively, compared to cells grown in a culture flask. There were no significant differences for the Pgp mRNA expression (Figure 4b). The complementary ddPCR analysis highlighted that the mRNA expression of TJ proteins was greater (for ZO-1 and CL-5 by 1.2-fold, and for OCL by a 1.4-fold change increase, respectively). A 1.2-fold decrease was noticed for the GLUT-1 transporter in the cells grown on the polyester membrane, while no difference was noticed for Pgp mRNA expression (Figure S3b).

#### 3.2.3. Immunostaining of TJ Proteins

A qualitative identification of the TJ proteins CL-5 and ZO-1 was obtained by immunostaining. ECs monolayer models were stained just after the permeability assay by directly incubating them with anti-CL-5 and anti-ZO-1 antibodies. Negative controls were used, and no false positive response was observed (data not shown). The resulting fluorescent images revealed the formation of a continuous ECs monolayer, still undamaged after a 75-min permeability assay. Furthermore, the correct localization of the TJ proteins was verified (Figure S4). A semi-quantitative analysis of TJs was also performed by measuring fluorescence intensities and then normalizing them according to the cell number. The results showed a similar tendency as for the q-PCR ones, both CL-5 and ZO-1 appeared to be more expressed in BMEC than in bEnd.3 cell monolayers (Figure S5).

### 3.3. In Vivo/In Vitro Correlation and Validation

Performance of the in vitro established models was compared to a PAMPA-BBB assay which mainly discriminates drug permeabilities according to their partition coefficient and is used as an early screening tool of large libraries of newly-synthetized compounds. To that extent, seven drugs presenting a wide range of physicochemical properties (Table 1) were tested in vitro on the three different models. Furthermore, the in vivo permeability of these drugs (log BB) was also determined in C57BL/6 mice.

**Figure 5:**
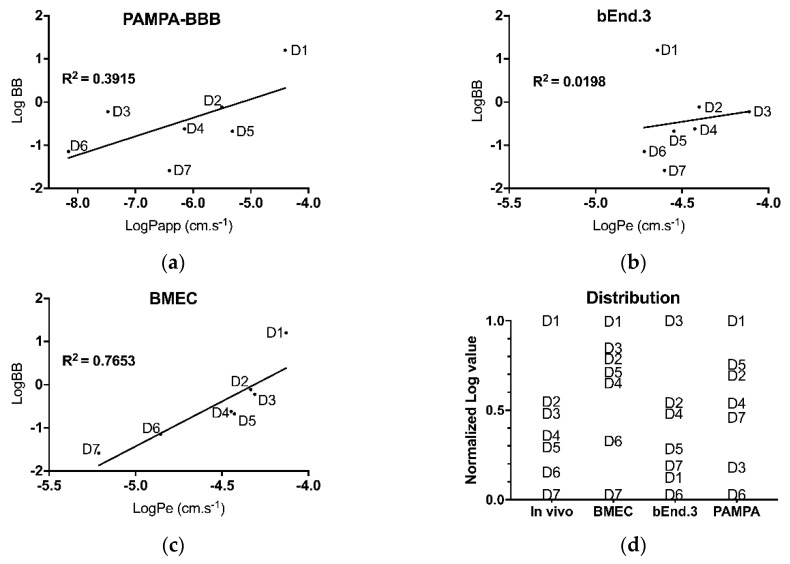
Linear regression plots for in vitro Pe–in vivo log BB correlation (IVIVC) using different models for seven drugs: (**a**) PAMPA-BBB, (**b**) bEnd.3 model and (**c**) BMEC. Pe measurements were realized on day 7. LogBB were quantify 2 h post drugs injection. (**d**) Distribution of drug ranking according to the selected model. D1: chlorpromazine; D2: midazolam, D3 caffeine, D4: theophylline, D5: verapamil, D6: atenolol, D7: tenoxicam. Each point indicates the average Log BB or Log Pe for a given drug (*n* ≥ 4).

First, Papp or Pe coefficients were calculated for each of the seven drugs, in each of the three in vitro models. Obtained values were then plotted against the logarithm value of the brain-to-blood concentration ratio (log BB) (Figure 5a–c).

For each model, a correlation coefficient (r^2^) was calculated by linear regression. This r^2^ value was used to assess the reliability of each model to predict in vivo permeability. The correlation coefficient for the PAMPA-BBB model was found to be r^2^ = 0.391 (Figure 5a) whereas for the bEnd.3 model, r^2^ = 0.019 (Figure 5b) and for BMEC model, r^2^ = 0.765 (Figure 5c).

Permeability values were then normalized to determine drug ranking from ‘best ability to cross the BBB’ to ‘worst ability to cross the BBB’, based on each model. The in vivo results were used as a reference and each in vitro model was challenged for its capacity to rank drugs in the same order as observed in mice. For the most lipophilic tested drug, i.e., chlorpromazine, only the BMEC and PAMPA-BBB models properly classified it as ‘best ability to cross the BBB’. As for the most hydrophilic drug, namely tenoxicam, only the BMEC model ranked it correctly as ‘worst ability to cross the BBB’. Globally, of the three in vitro models, only the BMEC model has ranked the 7 drugs close to that observed in vivo. Both the PAMPA-BBB and bEnd.3 models proved their inability to correctly discriminate these seven drugs (Figure 5d).

## 4. Discussion

### 4.1. Although Morphologically Identical, Primary and Immortalized Brain ECs Display Different Genomic Patterns

BBB primary cells are generally criticized because their isolation is expensive, laborious, and the resulting culture is often contaminated with other BBB cells, such as pericytes or astrocytes [28]. Those contaminated cells may be linked to an increase in experimentation variability. However, this issue is currently partially addressed since recent cell isolation protocols are easier to perform [29]. Moreover, the use of puromycin leads to an enriched culture in 5–7 days [27], as in the case of the primary ECs we isolated from the adult brains of C57BL/6 mice. The isolation process took six hours to perform on 10 to 30 mice, yielding around two million cells per 10 mice. The time-lapse from seeding the extracted capillaries to observing a confluent ECs monolayer was maximum 7 days, only one third of the time needed to establish a Caco-2 monolayer [30]. Despite their longer time to confluency, these latter cells are currently widely used in pharmaceutical R&D for drug screening.

On the other hand, immortalized brain endothelial cell lines are readily available and their fast proliferation rates lead to a confluent cell monolayer in 2 to 4 days. Immortalized cells theoretically preserve most of their original characteristics, but some are diminished or even lost when passaged. Herein, we used the bEnd.3 cells, an immortalized cell line obtained by infecting primary cultures of brain ECs from BALB/c mice with the middle T-expressing N-TKmT retrovirus [31]. The bEnd.3 cell line was proposed as a model for the evaluation of BBB function [28], especially the paracellular barrier [32], and as a BBB model for drug uptake and transport studies [33].

In order to establish the validity of the studied models, we first examined cell features for known EC morphology and characteristics. The microscopic observation showed similar spindle-shape morphology, typical of ECs [34] for both primary and bEnd.3 cells (Figure S1). Cells morphology was important to monitor since ECs could change their shape following modifications of their phenotype due to culture conditions. As the BMEC model established in this work was designed for drug screening, evaluation of the presence of main MBRT and TJ proteins, implicated in drug passage across the BBB, was mandatory.

Based on comparative qPCR analyses, mRNA gene expression of Pgp, BCRP, GLUT-1, LAT-1, ZO-1, OCL, and CL-5 was confirmed in BMEC and bEnd.3 cells, both grown in their own culture medium. Relative expression of all tested genes, except BCRP, was statistically increased in BMECs (Figure 1a). As only the BMEC medium contained growth factors and puromycin, substances known to increase the expression of certain proteins [35], we compared the expression of the same genes from BMEC and bEnd.3 cells both grown in the richer BMEC medium. In that case, Pgp, BCRP, GLUT-1, LAT-1 and OCL relative expressions became even higher, in favor of the primary cells (Figure 1b). The lower mRNA expression of these genes in bEnd.3 cells, as compared to primary cells, stand out as an unavoidable transformation due to immortalization, as previously reported for other cell lines [36]. The Western blot analysis of TJ proteins performed by Watanabe et al., showed comparable tendencies, i.e., similar protein expression levels for OCL, an increased level for ZO-1, and a decrease for CL-5 when comparing primary mouse ECs with the bEnd.3 cell line [32]. However, primary cells used in their study were isolated from ICR mice, as opposed to C57BL/6 mice used in ours.

Pgp, an efflux protein transporter, is another BBB highly-expressed protein to be found in a valid BBB model. Pgp mRNA gene expression was more than 3-log increased in primary compared to bEnd.3 cells. To ascertain this yet unreported result, we performed a MTS viability assay in presence of increasing concentrations of puromycin, a Pgp substrate. Results showed that, only in BMEC, the Pgp transporter was effective enough to maintain a high cell viability, as opposed to bEnd.3 in which only 50% of cells survived after a 24-h exposure (Figure 2). Consequently, BMECs not only expressed more Pgp mRNA, but this mRNA was translated into a higher number of functional, active Pgp proteins present at the cell membrane.

### 4.2. Primary Cells Form a Tighter, More Impermeable Monolayer Model Than Immortalized Cells

Cells were turned into in vitro models when they were cultured on porous polymeric membranes separating two compartments (Transwell^®^ plates). When evaluating a BBB model, two basic characteristics must be retrieved: its electrical resistance, by calculating the TEER, and its Pe for paracellular markers [24]. Both parameters were therefore used to compare the effectiveness of the BMEC and the bEnd.3 models to form a tight monolayer.

First of all, TEER values globally reflect the monolayer integrity and the ability of cells to form TJ between them. From our protocol, the primary BMEC monolayer displayed a TEER value of about 80 ± 10 Ω × cm^2^ at day 7, using electrode chambers to measure the electrical resistance (Figure 3a). In order to establish its relevance, we sought to compare this value with those reported by others for similar models. Results were very disparate, ranging from 20–30 Ω × cm^2^ [29,37] to 140 Ω × cm^2^ [22], with most common values around 120–125 Ω × cm^2^ [38]. Those discrepancies could be linked to various time-to-confluency (from 5 to 7 days), electrode formats (chopstick-like, chambers or automated systems), insert surface (12- or 24-well plates), or cell seeding density. As for the bEnd.3 model, the observed TEER values were twice smaller (≈40 ± 4.5 Ω × cm^2^) than those of the BMEC model (Figure 3a). Once again, values retrieved from various research articles showed divergences, ranging from 17 Ω × cm^2^ [8] to 140 Ω × cm^2^ [39], with most of the reported values in the 20–40 Ω × cm^2^ range [22,34].

The electrical resistance measurement of static monolayer models is usually performed using voltmeters, equipped either with chopstick-like electrodes or electrode chambers, and more rarely continuous automated systems. The chopstick-like electrodes give higher and variable resistance values in comparison with electrode chambers resulting in smaller but steadier values [24]. Besides, such factors as seeding density [37], time-to-confluency, as well as medium composition [40], insert membrane surface and their commercial source [22] have been reported to impact TEER values. Accordingly, TEER values cannot be used alone as markers for model tightness and must be backed by permeability studies.

The BMEC model showed no statistical difference in Pe for the paracellular hydrophilic marker NaFl when compared with the bEnd.3 model (Figure 3b), as previously reported [32]. Additionally, the BMEC model showed statistically different permeability values when using either 376-Da NaFl or 150-kDa FITC-D, a higher molecular weight marker with lower permeability. On the contrary, no permeability was detected for bEnd.3 model for FITC-Dextran. This result was surprising as all other parameters tended to demonstrate that the BMEC model was tighter than the bEnd.3 one. Kutuzov et al., demonstrated that the permeability of hydrophilic molecules depended on their molecular weight [41] and that the endothelium negatively-charged glycocalyx was implicated in the process when the molecular weight of tested molecules was over 600 kDa [42]. The presence of the glycocalyx at the bEnd.3 cell surface [43], but less in BMECs [44], could have reduced the passage of the 150-kDa FITC-D in the bEnd.3 model, thus preventing its quantification. The extent of glycocalyx involvement in drug permeation through the BBB is, however, still unknown. This result shows a possible limitation of the BMEC model when used for the screening of large hydrophilic molecules. This point warrants further investigations.

As the setup of the in vitro models implied growing cells on polyester membrane inserts instead of plain polystyrene plates, we evaluated whether changes in mRNA gene expression for specific proteins occurred when shifting from one support material to another. Wuest et al., reported an impact of the support material on cell growth [22]. However, their study was limited to TEER values and Pe. In our case, we assessed the relative quantification of MBRT and TJs mRNA as well.

For the BMEC model, even if the transfer to the polyester membrane showed a statistically significant increase in the mRNA expression of CL-5 and ZO-1, the biological relevance was disputable (<2-fold increase, Figure 4a). For the bEnd.3 model, the mRNA expressions of CL-5 and ZO-1 were increased by 6.6 and 2.3-fold (Figure 4b). A previous study regarding the bEnd.3 model showed no change in the mRNA expression for those proteins when grown on permeable Transwell^®^ supports [28]. However, the study used bEnd.3 cells with passages between 25–35 and information on the Transwell^®^ membrane material was omitted. An interesting fact is that CL-5 level is not proportional with TEER values, as cells like Caco-2, VB-Caco-2 and MDCK-MDR1 do not express CL-5 but show high values of TEER [45]. However, CL-5 and ZO-1, along with OCL, are the hallmark of brain ECs, even if their implication in the BBB permeability is not fully understood. Furthermore, CL-5 and ZO-1 immunostaining (Figure S4) allowed confirming the accurate localization of these proteins found at the cell membranes and the cell-cell points sealing the ECs in a tight, continuous monolayer.

Apart from ZO-1 and CL-5 for the bEnd.3 model, all other mRNA expression levels were not significantly altered from a biological standpoint (0.5 < change factor < 2-fold). According to these results, mRNA quantification from flask-cultured cells already gave a fair indication of the established model. The change of support material appeared to have only a slight impact on the ability of both cell types to comply with adequate model properties. Nonetheless, based on electrical resistance and permeability assays, the primary BMEC model was found superior to the bEnd.3 one.

### 4.3. Primary Cells Allow a Better Predictability of Drug Permeation Across the BBB

In vitro models are extremely valuable tools in the early stage of CNS drug discovery. Although the scientific community agrees to their limited capacity to accurately reproduce the in vivo BBB properties, those models are needed because they allow a rapid screening between various molecules at a lower cost than animal studies. In this context, an in vitro mouse model displays a great advantage, compared to other animal models, of being readily paralleled with in vivo data found in numerous publications.

The ultimate step of this study was thus to test the permeability of seven drugs on three in vitro models and compare the results with in vivo data obtained in mice. The comparison was achieved on two levels: the conventional IVIVC based on the linear correlation between log BB and logPapp ou logPe and a second approach consisting in comparing the normalized ranking of the 7 drugs with their in vivo ranking based on normalized log BB.

The IVIVC results of the 3 in vitro models yielded quite unexpected observations. The PAMPA-BBB model was expected to be the worst one to correlate with in vivo data as this simple, acellular model can only assess passive diffusion. The best correlation coefficient (r^2^ = 0.765, Figure 5c) was achieved by BMEC model. However, the second best was the PAMPA-BBB model (r^2^ = 0.39, Figure 5a), known as a suitable acellular model to evaluate the passive permeability of small lipophilic drugs [12]. The least reliable model, in our conditions, was the bEnd.3 model (r^2^ = 0.019, Figure 5b). Due to its nature, the PAMPA-BBB assay results (logPapp) best correlated with drug logP values (r^2^ = 0.7570). When removing from the analysis the two drugs with the highest water solubilities, i.e. atenolol (D6) and theophylline (D4), the correlation was even greater (r^2^ = 0.9692). Thus, the model seemed to display an overall better prediction for lipophilic drugs. However, logBB did not correlate well with logP (r^2^ < 0.36), demonstrating that the presence of cells in the in vitro models was mandatory to correctly predict drug passage in vivo. The two cellular models, surprisingly, gave very dissimilar correlation with in vivo results.

Therefore, drug ranking was also analyzed to offer the better possible predictability (Figure 5d). The distribution pattern of the drug rankings appeared very comparable between normalized log BB and logPe. Nonetheless, the drug ranking was utterly wrong with the bEnd.3 model, as it was for the PAMPA-BBB model. On the contrary, the BMEC model alone was capable to discriminate between high-, moderate- and low-permeability drug groups. Based on the work of Vilar et al. [46], drugs can be divided into three distinct groups according to their log BB: CNS^++^ or CNS^+^ (both displaying CNS activity) when log BB ≥ 0.3 or −1 < log BB < 0.3, respectively, and CNS^-^ (no known CNS activity) when −1 ≤ log BB. Thus, D6 and D7 could be qualified as CNS^−^, D2 to D5 as CNS^+^ and D1 as CNS^++^. The BMEC model generated a correct ranking, distinguishing correctly between the most brain-permeable molecule (chlorpromazine, D1) and the two least brain-permeable molecules, namely atenolol (D6) and tenoxicam (D7). The four CNS^+^ drugs were also correctly ranked as moderately permeable, with D2/D3 (midazolam/caffeine) and D4/D5 (theophylline/verapamil) grouped as close pairs.

To the best of our knowledge, this study is the first to design and validate a primary mouse model, correlated with in vivo data obtained at the same time. Previously published by Nakagawa et al., the validation of a rat triple BBB model with 19 drugs yielded a r^2^ = 0.89 [23]. However, the in vivo data was obtained only after 5 min post drug injection. Another study reported the screening of 10 molecules on different in vitro models with various successes: a primary rat model (r^2^ = 0.79), a Caco-2 model (r^2^ = 0.6), a VB-Caco-2 model (r^2^ = 0.72) and a MDCK-MDR1 model (r^2^ = 0.77) [45]. This work correlated their results with the in vivo data reported by Nakagawa et al. [23].

At this point, a parallel comparison between this primary mouse model with promising models designed with iPCS should be made. Indeed, iPCS theoretically offer limitless cell access, where primary cells, regardless of their origin, should be used within a few passages. Yet, an optimized primary cell isolation yields to an >90% enriched culture in 7 days, compared with iPCS models, which require a few weeks and highly complex media to reach fully differentiated cell types. Nonetheless, the iPCS BBB models display higher TEER values and lower Pe, when compared to other in vitro models. The observed increase of monolayer tightness could be the result of the medium composition, which counts growth factors, drugs, neural inducers used for the reprogramming of cells back to the embryonic pluripotent state and for further differentiation into the target cell type [18]. Unfortunately, as for the characterization other than tightness and protein expression of the iPCS BBB in vitro models, no IVIVC was yet established. However, this correlation is needed to assess the ability of the designed models to accurately screen and rank drugs. As iPCS models still need to be studied and optimized, primary ones should be currently preferred for drug screening studies.

Limitations of this study involve the number of tested molecules, which could be increased, and the fact that the log BB was calculated in a standardized manner, i.e., 2 h post-injection. This latter point could be improved by taking into account pharmacokinetics parameters for each individual drug. For instance, different elimination half-lives could contribute differently to brain accumulation [47]. A way to eliminate such variability, especially for drugs under development, would be to quantify the brain-blood ratio at 3 different time points, in order to encompass quantification at brain equilibrium half-life [48]. Another limitation to the model presented herein is its simplistic composition of only ECs. Adding astrocytes and pericytes could increase the overall predictability of the model, as both cell types are known to increase the barrier tightness. However, from our point of view, this is detrimental to the model ease of use. This simple BMEC model also offers the unique possibility to easily study the underlying mechanisms involved in the passage of drug molecules into the brain compartment. However, data may be tempered as they are produced on a rodent cell-based model and may not be transposable as is to human studies. In addition, whenever in need for a predictive tool for BBB passage of small molecules, the BMEC model appears far more effective than the bEnd.3 model and the conventional PAMPA-BBB model.

## 5. Conclusions

In the present study, we compared two in vitro cellular models of the BBB with in vivo permeability data. To achieve this comparison, we examined those models on three different stages. At the cellular level, although morphologically very similar, the BMEC model displayed higher levels of junctional and transporter proteins than the immortalized bEnd.3 cells, especially the crucial P-gp efflux protein. When assembled into a two-compartment model, the BMEC model demonstrated both higher TEER values and more discriminating capabilities than the bEnd.3 model. Taken together, these favorable features translated into a better IVIVC for a panel of seven drugs displaying a wide range of blood-brain distribution.

An ideal BBB model intended for drug screening should enable low-cost, rapid, and reproducible assessment of a drug capability to overcome or not the BBB’s natural barrier characteristics. Among all proposed static cell models, the mouse BMEC model, based on 0.33-cm^2^ Transwell^®^ inserts, requires less cell material, and therefore fewer animal sacrifices, than in vivo permeability studies. The mouse remains the most polyvalent and well-studied animal model in research studies. Given the wide variety of specific mouse knockout-strains and the well-studied mouse genome, the design of a validated BBB model from mouse primary brain cells appears an invaluable asset to early-phase drug screening. The BMEC model evaluated in this study, although not ideal in all aspects, can nevertheless provide precious information on BBB permeability and is therefore a worthwhile tool in CNS drug discovery.

## Figures and Tables

**Table 1:** List of the drugs selected for the validation of BMEC and bEnd3 monolayer models.

Abbreviation	Name	MW (g/mol)	log P ^1^
D1	Chlorpromazine hydrochloride (Enzo life Sciences, Lausanne, Switzerland USA)	355.3	5.41
D2	Midazolam (TRC, Lowell, MA, USA)	325.8	4.33
D3	Caffeine, anhydrous (Medisca, Montréal, QC, Canada)	194.2	−0.07
D4	Theophylline, anhydrous (Medisca, QC, Canada)	180.2	−0.02
D5	Verapamil hydrochloride (Sigma-Aldrich, Oakville, ON, Canada)	491.1	3.79
D6	Atenolol (Acros Organics, Springfield, New Jersey, USA)	266.3	0.16
D7	Tenoxicam (Alfa Aesar, Haverhill, MA, USA)	337.4	1.90

^1^ Information source: PubChem.

**Table 2:** Summary of mass spectrometry conditions.

**HPLC**	**Agilent 1100 Series**			
MS/MS	MDS Sciex 4000 Qtrap			
Software	Analyst^®^ (v1.6.2)			
Ionisation source, mode	Turbo electrospray, positive ionisation			
Scan mode	Multiple reaction monitoring (MRM)			
Analyte parameters	**Compounds**	**DP (V)**	**MRM**	**CE (eV)**
	Verapamil	110	455.3 > 165.0	60
	Midazolam	90	326.2 > 291.1	42
	Chlorpromazine	65	319.2 > 86.0	28
	Caffeine	90	181.1 > 124.2	28
	Atenolol	41	267.1 > 145.0	45
	Theophilline	70	194.1 > 138.2	27
	Tenoxicam	71	337.3 > 121.0	33
	Metochlopramide (ISTD)	70	300.1 > 184.3	44
Source parameters	Gas temp (°C)		550	
	Gas flow (L/min)		50	
	Curtain gaz (psi)		25	
	Capillary (V)		5500	
Mobile phase	Composition		A: 0.1% FA+ H_2_O	
			B: 0.1% FA + ACN	
	Gradient		2 to 98% B in 3.5 min	
Flow rate	0.75 mL·min^−1^			
Column temperature	45 °C			
Injection volume	4 µL			
Injection temperature	5 °C			
Column	YMC-Pack ODS-AQ, (50 × 3.0 mm, 5 µm)			

**Table 3:** Primers sequences and parameters used in this study for the analysis of the mRNA expression of main transporters and tight junction proteins.

Target Protein/Abbreviation/Gene Symbol	Function	Reference Sequence	Forward/Reverse Primers
P-glycoprotein/Pgp/Abcb1a	Efflux transporter	NM_011076.2	gggcatttacttcaaacttgtca/tttacaagcttcatttcctaattcaa
Breast cancer resistance protein/BCRP/Abcg2	Efflux transporter	NM_011920.3	aggtctggaaaaagtagcagattc/ctccatccctatgcttgtcc
Glucose transporter-1/GLUT-1/Slc2a1	Uptake transporter	NM_011400.3	gtatcctgttgcccttctgc/tcgaagcttcttcagcacac
L-type amino acid transporter/LAT-1/Slc7a5	Uptake transporter	NM_011404.3	tcagcttcttcaactggctgt/ggagggccagattcacct
Zonula occludens-1/ZO-1/Tjp1	Tight junction	NM_009386.2, NM_001163574	cgcggagagagacaagatgt/gaagcgtcactgtgtgctgt
Occludin/OCL/Ocln	Tight junction	NM_008756.2	gtccgtgaggccttttga/ggtgcataatgattgggtttg
Claudin-5/CL-5/Cldn5	Tight junction	NM_013805.4	ttaaggcacgggtagcactc/atgttggcgaaccagcag
Hypoxanthine-guanine Phosphoribosyltransferase/HPRT/Hprt	Endogenous protein	NM_013556.2	tcctcctcagaccgctttt/cctggttcatcatcgctaatc

**Table 4:** Drug formulation (in vivo and in vitro) and dosage (in vivo) used for the permeability studies.

Molecule Name	Molecule Formulation (%)	Administered Dose (mg/kg) ^1^
Chlorpromazine HCl	PEG 400/Water (30:70)	2.0
Midazolam	PEG 400/Water (30:70)	3.0
Caffeine	NaCl 0.9% (100)	12.4
Theophylline	DMSO/PEG 400/Water (10:30:60)	27.2
Verapamil HCl	PEG 400/Water (30:70)	1.52
Atenolol	DMSO/PEG 400/NaCl 0.9% (10:30:60)	10.0
Tenoxicam	DMSO/Solutol HS15 10% in PBS (10:90)	5.0

^1^ The administered dose was calculated as 20% of the LD_50_ injected IV in mice and then adjusted if necessary.

## Supplementary Materials

The following are available online at https://www.mdpi.com/1999-4923/11/11/587/s1, Figure S1: Morphology of (a) murine primary brain endothelial cells (BMEC, passage 1) and (b) murine bEnd.3 endothelial cell line (bEnd.3, passage 15) analyzed by optical inverted microscopy; Figure S2: Differences in mRNA expression fold-change between BMEC (red) and bEnd.3 cell line (blue), both grown in specialized BMEC medium; Figure S3: Differences in gene expression fold-change between (a) BMEC and (b) bEnd.3 cells grown on a polyester membrane insert filter (red) compared to cells grown on plastic cell culture flask (blue); Figure S4: Immunocytochemistry of tight junctions (TJs) on BMECs and bEnd.3 in vitro cell monolayer models. The 7-day old cell monolayers were directly fixed on the insert membrane and then incubated with Alexa 488-labelled (green) anti-ZO-1 and anti-CL-5 antibodies. DAPI staining (blue) was used to show nuclei. Figure S5: Relative quantification of the tight junction proteins (TJs), claudin-5 (CL-5) and zonula occludens 1 (ZO-1) from immunocytochemistry on in vitro BMECs and bEnd.3 cell monolayer models. The 7-day old cell monolayers were directly fixed on the insert membranes and then incubated with Alexa 488-labelled (green) anti-ZO-1 and anti-CL-5 antibodies. Fluorescence intensities were normalized according to cell numbers. *n* = 4. *T*-test, * *p* value < 0.05, *** *p* value < 0.001. Raw and analysis data available at (click on the link); Figure 1: https://figshare.com/s/cae07e1e56182b800aae; Figure 2: https://figshare.com/s/daf93a0fc9bbbc247660; Figure 3: https://figshare.com/s/5fb261b55e95ff346874; Figure 4: https://figshare.com/s/94e05ad165ed41cd26b6; Figure 5: https://figshare.com/s/2fab0c50e0a2f9e1e0f6.
