# Erratum: Puscas, I.; et al. IVIVC Assessment of Two Mouse Brain Endothelial Cell Models for Drug Screening. *Pharmaceutics* 2019, *11*, 587

**DOI:** 10.3390/pharmaceutics12060514

**Published:** 2020-06-04

**Authors:** Ina Puscas, Florian Bernard-Patrzynski, Martin Jutras, Marc-André Lécuyer, Lyne Bourbonnière, Alexandre Prat, Grégoire Leclair, V. Gaëlle Roullin

**Affiliations:** 1 Faculty of Pharmacy, Université de Montréal, CP6128 Succursale Centre-ville, Montreal, QC H3C 3J7, Canada; ina.puscas@umontreal.ca (I.P.); florian.bernard@umontreal.ca (F.B.-P.); martin.jutras@umontreal.ca (M.J.); 2 Department of Neuroscience, Faculty of Medicine, Université de Montréal and Centre de Recherc and du CHUM (CRCHUM), Montréal, QC H2X 0A9, Canada; marc-andre.lecuyer@mail.mcgill.ca (M.-A.L.); lyne.bourbonniere.chum@ssss.gouv.qc.ca (L.B.); a.prat@umontreal.ca (A.P.); 3 Centre for Biostructural Imaging of Neurodegeneration, Institute for Multiple Sclerosis Research and Neuroimmunology, University Medical Center Göttingen, 37075 Göttingen, Germany

The authors wish to make the following corrections to this paper [1]:

The version of Figure 3a that was uploaded with the manuscript was based on a calculation error, whereas the publication text referred to the correct version of Figure 3a. After the publication of this work, we noted the mistake and issued an erratum on the *y*-axis. Figure 3a has now been corrected in this erratum. 

The authors would like to apologize for any inconvenience caused to the readers by these changes.

## Figures and Tables

**Figure 3:**
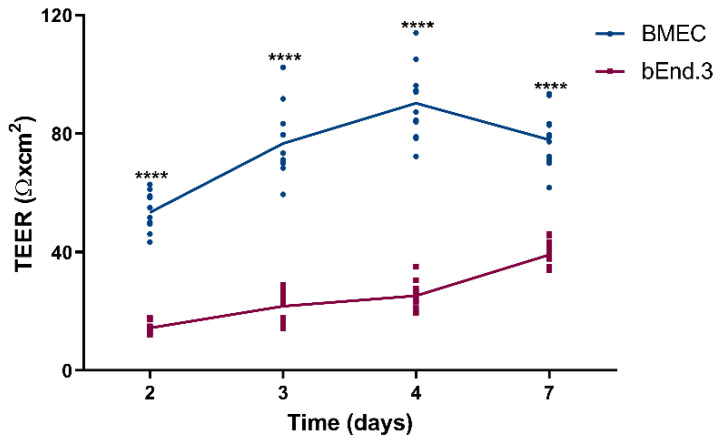
(**a**) Transendothelial electrical resistance (TEER, expressed as Ω × cm^2^) and (**b**) endothelial Pe for sodium fluorescein (NaFl) and FITC-dextran (Pe, expressed in cm/s) of the blood–brain barrier models built from mouse primary brain endothelial cells (BMEC, blue) and from mouse brain endothelial cell line (bEnd.3, red) at day 7. All data are presented as means ± SD (*n* = 12 for TEER, *n* = 4 for Pe). Statistical analysis: unpaired t test with Welch’s correction (ns: *p* ≥ 0.0332, **** *p* < 0.0001, ND- not detected).
