# Supplementary material for: IVIVC Assessment of Two Mouse Brain Endothelial Cell Models for Drug Screening
Source: Pharmaceutics. 2019 Nov 8;11(11):587. doi: 10.3390/pharmaceutics11110587 (PMC6920823; doi:10.3390/pharmaceutics11110587)
Supplement: Supplementary file 1 [file pharmaceutics-11-00587-s001.pdf]

pharmaceutics

Supplementary Materials: IVIVC Assessment of Two Mouse Brain Endothelial Cell Models for Drug Screening

Ina Puscas, Florian Bernard-Patrzynski, Martin Jutras, Marc-André Lécuyer, Lyne Bourbonnière, Alexandre Prat, Grégoire Leclair and V. Gaëlle Roullin

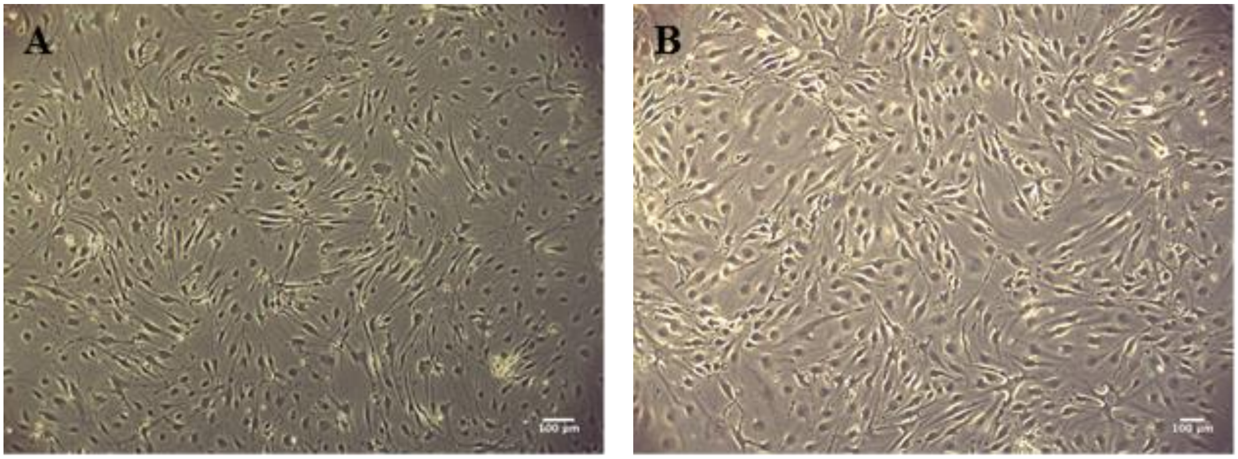

Figure S1. Primary brain endothelial cells (BMEC, passage 1) and (B) murine bEnd.3 endothelial cell line (bEnd.3, passage 15) analyzed by optical inverted microscopy. Images were captured at a magnification of × 20. Scale bar: 100 μm.

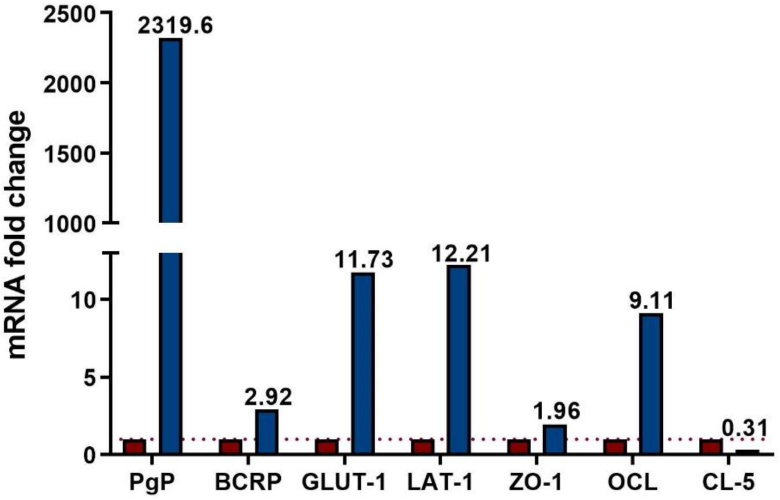

Figure S2. Differences in mRNA expression fold-change between BMEC (blue) and bEnd.3 cell line (red), both grown in specialized BMEC medium. Gene expression was quantified by ddPCR, using HPRT mRNA expression as the internal control gene and the bEnd3 cell line as the calibrator (n =3).

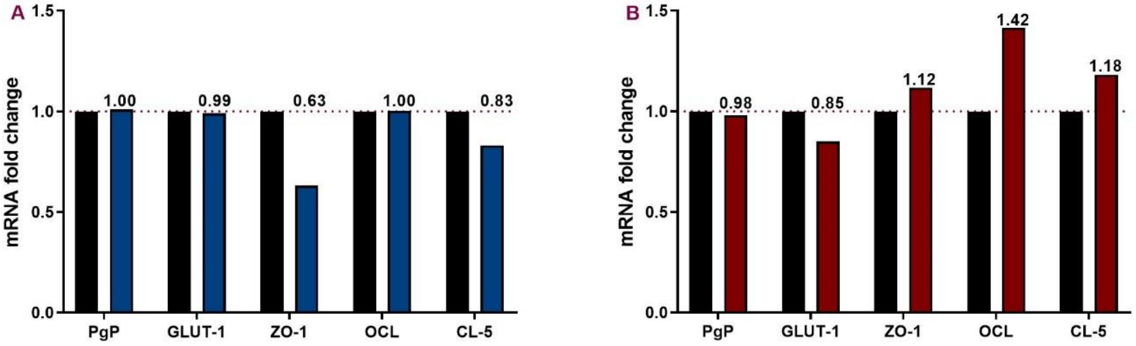

Figure S3. Differences in gene expression fold-change between (A) BMEC and (B) bEnd.3 cells grown on a polyester membrane insert filter pre-coated with collagen type IV (black) compared to cells grown on plastic cell culture flask pre-coated with collagen type IV (blue-BMEC/red-b.End3). Gene expression was quantified by ddPCR, using HPRT mRNA expression as the internal control gene and cells grown on the plastic cell culture flask as the calibrator (n = 3).

pharmaceutics

MDPI

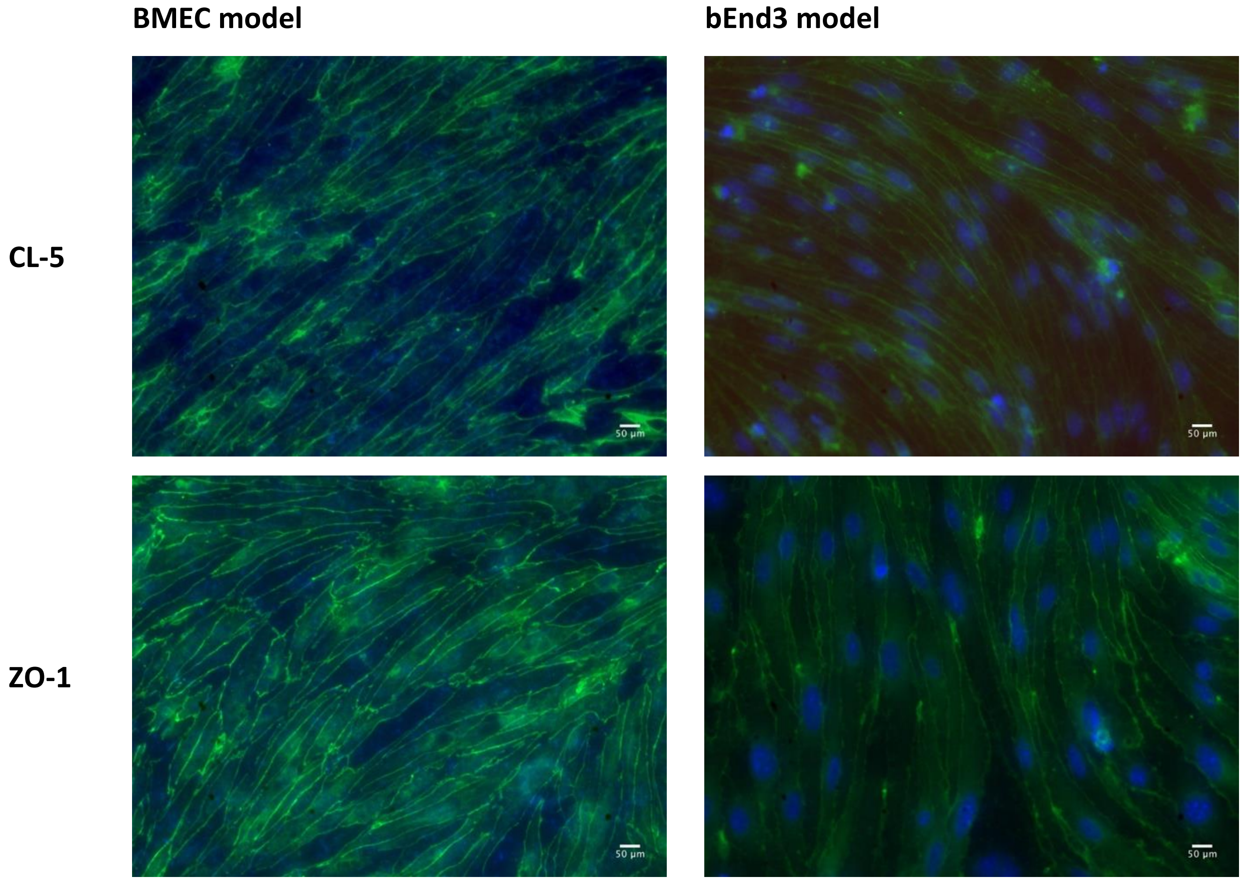

Figure S4. Immunocytochemistry of tight junctions (TJs) on BMECs and bEnd.3 *in vitro* cell monolayer models. The 7-day old cell monolayers were directly fixed on the insert membrane and then incubated with Alexa 488-labelled (green) anti-ZO-1 and anti-CL-5 antibodies. DAPI staining (blue) was used to show nuclei. Magnification in all images: ×20. Scale bar 50 μm. Representative data of n=4.

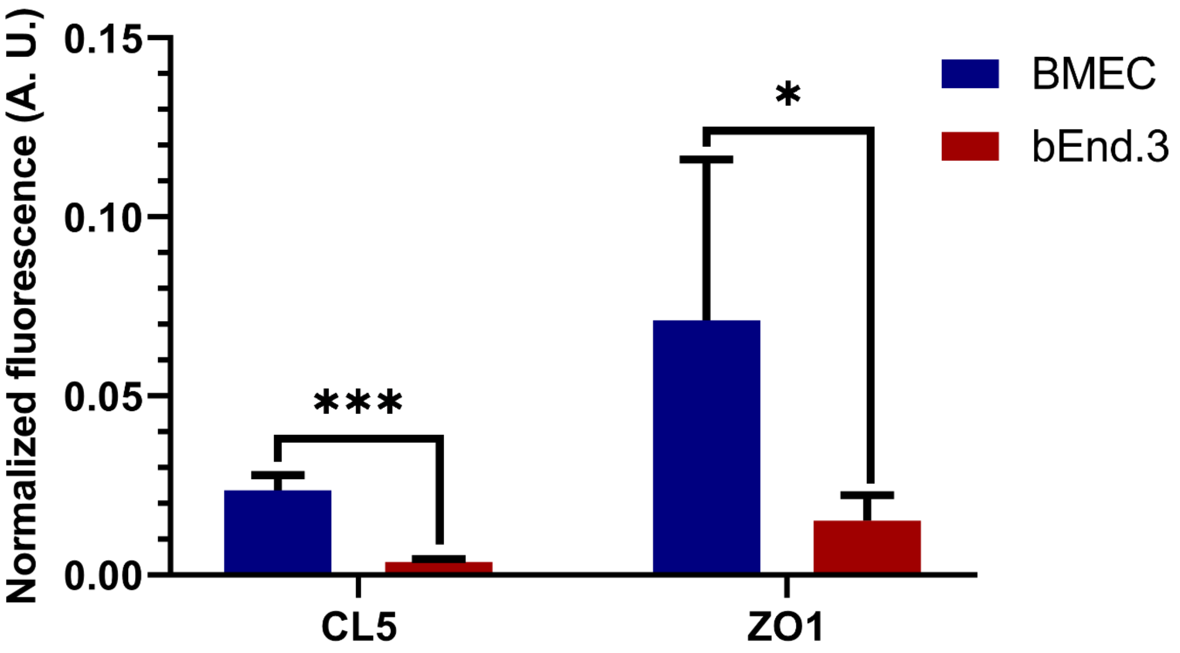

Figure S5. Relative quantification of the tight junction proteins (TJs), claudin-5 (CL-5) and zonula occludens 1 (ZO-1) from immunocytochemistry on *in vitro* BMECs and bEnd.3 cell monolayer models. The 7-day old cell monolayers were directly fixed on the insert membranes and then incubated with Alexa 488-labelled (green) anti-ZO-1 and anti-CL-5 antibodies. Fluorescence intensities were normalized according to cell numbers. n = 4. T-test, * : p value < 0.05, *** : p value < 0.001.
